# Early and Long-Term Clinical and Echocardiographic Outcomes of Sutureless vs. Sutured Bioprosthesis for Aortic Valve Replacement

**DOI:** 10.3390/jcdd10050224

**Published:** 2023-05-22

**Authors:** Aleksander Dokollari, Gianluca Torregrossa, Gianluigi Bisleri, Ali Fatehi Hassanabad, Michel Pompeu Sa, Serge Sicouri, Altin Veshti, Edvin Prifti, Beatrice Bacchi, Francesco Cabrucci, Basel Ramlawi, Massimo Bonacchi

**Affiliations:** 1 Department of Cardiac Surgery, Lankenau Heart Institute, Wynnewood, PA 19096, USA; dokollaria@mlhs.org (A.D.);; 2 Department of Cardiac Surgery Research, Lankenau Institute for Medical Research, Wynnewood, PA 19096, USA; 3 St. Michael’s Hospital, Toronto, ON M5B 1W8, Canada; 4 Section of Cardiac Surgery, Department of Cardiac Sciences, Libin Cardiovascular Institute, Cumming School of Medicine, Calgary, AB T2N 4N1, Canada; 5 Cardiac Surgery Department, Mother Teresa Hospital, University of Tirana, 1000 Tirana, Albania; 6 F.U. Clinical and Experimental Medicine, University of Florence, 50134 Florence, Italy

**Keywords:** sutureless, sutured bioprosthesis, perceval, echocardiographic outcomes, clinical outcomes, long-term outcomes

## Abstract

**Objective**: The goal of this manuscript is to compare clinical and echocardiographic outcomes of patients undergoing aortic valve replacement (AVR) with Perceval sutureless bioprosthesis (SU-AVR) and sutured bioprosthesis (SB). **Methods**: Following the PRISMA statement, data were extracted from studies published after August 2022 and found in PubMed/MEDLINE, EMBASE, CENTRAL/CCTR, ClinicalTrials.gov, SciELO, LILACS, and Google Scholar. The primary outcome of interest was post-procedural permanent pacemaker implantation, and the secondary outcomes were new left bundle branch block (LBBB), moderate/severe paravalvular leak (PVL), valve dislocation (pop-out), need for a second transcatheter heart valve, 30-day mortality, stroke, and echocardiographic outcomes. **Results**: Twenty-one studies were included in the analysis. When SU-AVR was compared to other SB, mortality ranged from 0 to 6.4% for Perceval and 0 to 5.9% for SB. Incidence of PVL (Perceval 1–19.4% vs. SB 0–1%), PPI (Perceval 2–10.7% vs. SB 1.8–8.5%), and MI (Perceval 0–7.8% vs. SB 0–4.3%) were comparable. In addition, the stroke rate was lower in the SU-AVR group when compared to SB (Perceval 0–3.7% vs. SB 1.8–7.3%). In patients with a bicuspid aortic valve, the mortality rate was 0–4% and PVL incidence was 0–2.3%. Long-term survival ranged between 96.7 and 98.6%. Valve cost analysis was lower for the Perceval valve and higher for sutured bioprosthesis. **Conclusions**: Compared to SB valves, Perceval bioprosthesis has proved to be a reliable prosthesis for surgical aortic valve replacement due to its non-inferior hemodynamics, implantation speed, reduced cardiopulmonary bypass time, reduced aortic cross-clamp time, and shorter length of stay.

## 1. Introduction

The advent of sutureless valves for aortic valve replacement (SU-AVR) has enabled surgery in patients who would otherwise not be surgical candidates due to frailty or prolonged surgical procedures.

SU-AVR self-expanding Perceval aortic bioprosthesis (LivaNova Group, Milan, Italy) was developed to combine the advantages of transcatheter aortic valve replacement (TAVR) [1] procedure, allowing for fast implantation with no need for suturing, with the benefits of a conventional surgical approach owing to the possibility of removing the native valve along with calcifications. The benefits of SU-AVR extend to severely calcified aortic annuli not amenable to standard sutured bioprosthesis implantation due to complications, including paravalvular leaks and prosthesis detachment, necessitating conversion to the Bentall procedure.

Previous reviews have proven the benefits and pitfalls of SU-AVR over sutured bioprosthesis for aortic valve replacement (SAVR) [2,3,4] evidencing patients’ benefits from the procedure. In addition, the PARTNER clinical trials [5,6,7], the SURTAVI trial [8], and other meta-analyses [9,10] have evidenced the non-inferiority of TAVR vs. SAVR. In addition, other outcomes of the valve include improved hemodynamics, a self-expanding radial force, usage in hostile roots, enhanced surgical and recovery speed, and enabling minimally invasive cardiac surgery procedures. However, many points deserve to be highlighted, such as the impact of permanent pacemaker implantation (PPI) after SAVR, the use of SU-AVR in patients with bicuspid aortic valves (BAV), valve costs analysis, as well as echocardiographic outcomes.

A major debate exists on long-term clinical and echocardiographic outcomes in patients undergoing SU-AVR. In this context, the three major points of discussion relate to (a) the time to degeneration of bioprosthetic leaflets after valve implantation, (b) the small aortic annuli outcomes after valve implantation, and (c) the impact of more than mild paravalvular regurgitation on long-term outcomes. While previous reviews have already demonstrated non-inferior short- and mid-term outcomes of SU-AVR, compared to sutured bioprosthesis (SB), long-term outcomes have yet to be fully established in the medical literature and deserve more consideration [9].

The goal of this review is to highlight the main target points covered by clinical trials and observational clinical studies and raise a point of discussion for further expansion of the use of SU-AVR.

## 2. Material and Methods

**Figure 1:**
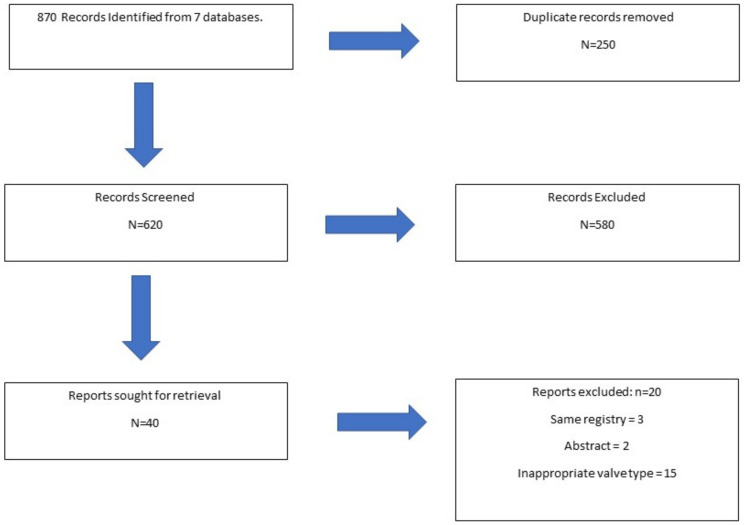
Flowchart of Patient Selection Process.

This review was carried out in accordance with the Preferred Reporting Items for Systematic Reviews and Meta-Analyses (PRISMA) guidelines (Figure 1) [10]. The following databases were searched for studies meeting our inclusion criteria and published by 28 February 2023: PubMed/MEDLINE, Embase, SciELO, LILACS, CCTR/CENTRAL, Google Scholar, and grey literature. We searched for the following terms: [“Heart Valve Prosthesis Implantation” OR “rapid-deployment aortic valve” OR “sutureless aortic valve” OR “Perceval” NOT “Enable”] AND [‘’Sutured versus Sutureless” OR ‘’Bioprosthesis versus Sutureless”]. The following steps were taken for study selection: (1) the identification of titles of records through database search; (2) the removal of duplicates; (3) the screening and selection of abstracts; and (4) the assessment for eligibility through full-text papers. Data are available upon reasonable request.

### 2.1. Inclusion Criteria

**Figure 2:**
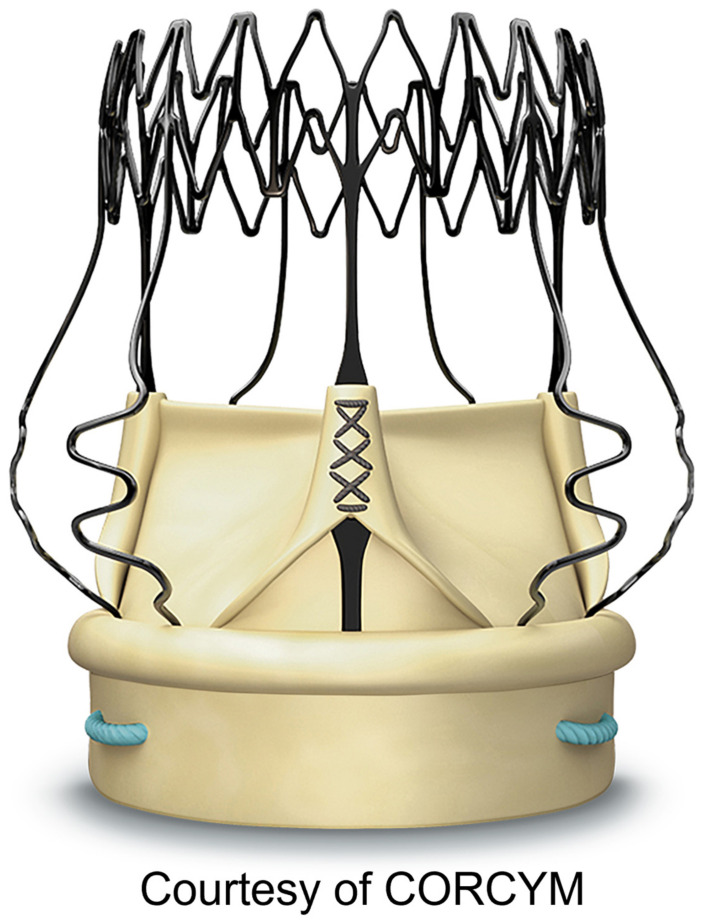
Perceval Bioprosthesis.

Studies were included if any of the following criteria were met: (1) reported outcomes of Perceval compared with other heart valve prostheses or procedures; (2) reported analysis of complications using Perceval valve (Figure 2); (3) reported off-label experience; and (4) reported learning curve analysis.

### 2.2. Exclusion Criteria

Studies were excluded if any of the following criteria were met: (1) reported outcomes of exclusively other SU-AVR [11,12,13,14,15]; (2) grouped outcomes of Perceval with other prostheses in the same cohort [16,17,18,19]; (3) not published in the English language; (4) not published in a peer-reviewed journal; (5) was a conference abstract [20,21,22,23]; and (6) case reports.

### 2.3. Data Collection

Data collection was conducted on 8 March 2023. One author (AD) screened the articles and reviewed them three times. The results were reviewed by another author (SS). Discrepancies were arbitrated by the third author to achieve consensus (MB). The primary reported outcomes of the study included (a) clinical trials outcomes investigating SU-AVR; (b) SU-AVR vs. other stented bioprostheses (c) SU-AVR in bicuspid aortic valves; (d) long-term outcomes of SU-AVR (valve durability); and (e) hospital costs.

### 2.4. Surgical Technique for Perceval Sutureless Valve Implantation

The most performed surgical approach for SU-AVR implantation is full sternotomy. Following heparin administration, standard central aortic and right atrial venous cannulation are initiated. After the institution of cardiopulmonary bypass (CPB), the aorta is cross-clamped and antegrade and/or retrograde cardioplegia is delivered. Aortotomy is performed, and the aortic valve is removed, with care taken for adequate removal of annular calcification and debridement. Therefore, the valve is implanted at the annulus level through three guiding stitches that are later removed, and the valve is ballooned at 4 atmospheres. After correct valve deployment and testing of the valve, the aorta is closed in standard fashion. Surgical centers with advanced expertise in minimally invasive cardiac surgery [8] find SU-AVR to be suitable for minimally invasive aortic valve replacement with either ministernotomy or right minithoracotomy.

## 3. Results

After excluding duplicates and non-eligible studies, 21 studies were included in the analyses.

### 3.1. Sutureless vs. Sutured Bioprosthesis

Nine retrospective and prospective clinical studies were included in the final analysis with 639 patients in the Perceval group and 1636 in the SB group (Table 1) [24,25,26,27,28,29,30,31]. Compared to other SB valves, mortality ranged from 0 to 6.4% for Perceval and 0 to 5.9% for SB. The incidence of a paravalvular leak (PVL) (Perceval 1–19.4% vs. SB 0–1%), PPI (Perceval 2–10.7% vs. SB 1.8–8.5%), stroke (Perceval 0–3.7% vs. SB 1.8–7.3%), and MI (Perceval 0–7.8% vs. SB 0–4.3%) were comparable.

### 3.2. Perceval in Bicuspid Native Aortic Valves

Six retrospective clinical studies with 157 patients were included in the final analysis (Table 2) [33,34,35,36,37]. The mortality rate was (0–4%), PVL (0–2.3%), stroke (0–7.6%), MI = 0%, PPI (0–7%), and aortic cross-clamping time was 39 ± 13 to 45.9 ± 14 min. Cardiopulmonary bypass time ranged between 54.5 ± 4.4 and 80 min.

### 3.3. Echocardiographic Outcomes

Echocardiographic data were collected from previously described studies (Table 3). Effective orifice area (EOA) upon hospital discharge ranged between 1.4 ± 0.4 and 1.56 ± 0.37 cm^2^. At 6-month (1.5 ± 0.3 to 1.5 ± 0.4 cm^2^), 1-year (1.5 ± 0.3 to 1.6 ± 0.4 cm^2^), and 2-year follow-ups (1.51 ± 0.26 to 1.7 ± 0.5 cm^2^), there were no significant changes. Mean and peak transvalvular gradients at discharge and up to 2-year follow-up did not significantly change (see Table 4 and Table 5).

### 3.4. Hospital Costs Outcomes

Three studies were included in the cost analysis. Hospital costs outcomes ranged in US dollars from $12,825 for SU-AVR and $13,543 for SB (Table 6).

## 4. Discussion

### Summary of Findings

(1) SU-AVR had a lower incidence of in-hospital complications and overall mortality when compared to SB.
(2) SU-AVR had the lowest hospital costs when compared to SB bioprosthesis.

## 5. Comments

This manuscript highlighted the most up-to-date outcomes from clinical studies, including the benefits and pitfalls of SU-AVR over SB for aortic valve replacement. In this context, we reported short- and long-term clinical and echocardiographic outcomes. In addition, we also reported the overall hospital costs for each of the valves. Based on the findings from this study, we hypothesize that patients and surgeons can benefit from these outcomes by aiding in the surgical decision process based on the individual patient risk profile.

### 5.1. Outcomes of Sutureless Valves

SU-AVR have made a significant advancement in the last decade and its design has been accepted as the preferred treatment of choice for patients with aortic valve disease who qualify for aortic valve replacement. In addition, TAVR has proven its non-inferiority when compared to other SB [46,47,48]. The strongest points of these valves include (a) the non-inferior hemodynamics outcomes; (b) a friendly implant in hostile annulus environments, such as endocarditis and reoperations; and (c) facilitating future valve-in-valve TAVR as sinus struts protect coronary ostia from obstruction and Nitinol cage expandable. In this study, we found and pointed out important clinical and procedural outcomes when SU-AVR is compared to other bioprostheses. We found that outcomes such as stroke, PPI, PVL, and echocardiographic reports are non-inferior to SU-AVR when compared to SB [6,7,8]. However, a future clinical trial comparing SB and SU-AVR will give more insight into the right choice of patient. When compared to other SB, the latest revealed higher CPB and aortic cross-clamp (AXC) time, higher incidence of stroke rate, and bleeding. The increased incidence of PPI in the Perceval group when compared to SB remains a burden and is mainly operator depended [46,49,50,51,52]. In this context, the learning curve plays a major role and the SB has been used for a longer period: therefore, the operator is more experienced in valve implantation.

### 5.2. Long-Term Clinical Outcomes

Long-term clinical outcomes reported an overall cardiac death incidence of 1.4–3.3%, a valve explant incidence of 0–1.5%, an incidence of paravalvular leak of 0–1% and a stroke incidence of 0–0.8%. In addition, risk predictors for SU-AVR that impact all-cause death included female sex [53]. On the other hand, SB outcomes at 5-year follow-up have shown an overall incidence of cardiac death of 2–2.6%, repeat intervention of 3–3.7%, and structural valve deterioration of 1–1.3% [32]. In addition, risk predictors for all-cause mortality include age, creatinine level, presence of CAD, and NYHA class [54]. This review highlights long-term clinical outcomes, including repeat intervention, cardiac death, incidence of stroke, and major paravalvular leaks.

### 5.3. Long-Term Echocardiographic Outcomes

Long-term echocardiographic outcomes of SU-AVR evidenced a preserved EF of around 60%, a mean transvalvular gradient of 8.8–9.3 mmHg, and an EOA of 1.8. Echocardiographic risk predictors for all-cause death in SU-AVR included left ventricle dysfunction of grade 3 [32]. Other studies using SB have shown an EF of 62% and a mean gradient of 20.6 mmHg [55], while risk predictors for death included the E/e’ index. This review is the largest study describing long-term echocardiographic outcomes in medical literature, providing new insights into outcomes, including transvalvular gradients and EOA.

### 5.4. Reported Cost Outcomes

The reported cost outcomes of SU-AVR are lower compared to SB. In this context, patients in developing countries have a higher incidence of rheumatic aortic valve disease, while hospitals have limited budgets. Therefore, SU-AVR satisfies both criteria, including hostile aortic roots after rheumatic disease and lower economic costs compared to other bioprostheses. However, these outcomes are difficult to measure due to different hospital costs among different countries and the annual currency inflation.

### 5.5. Comparison with Other Literature Reviews

When compared to the study by Powell et al. [9], this study review provides new insights into long-term echocardiographic and clinical outcomes. In this context, outcomes from this review provide clear answers to questions, such as what is the reintervention rate in patients undergoing SU-AVR? Can sutureless valves be removed if reintervention is necessary? What is the long-term evolution of transvalvular gradients? How does post-operative paravalvular leak impact long-term prognosis?

### 5.6. Comparison with Our Previous Study

When compared to our previous review, this study provides an update on short- and long-term outcomes after SU-AVR implantation, including an 8-year follow-up clinical study in patients undergoing SU-AVR replacement.

This study provides an update of the literature review on bicuspid valves and on short- and long-term outcomes when compared to our previous publication [2]. Once more, this review confirms the good clinical outcomes of the Perceval valve from the literature. In addition, a meta-analysis done by our group [4] evidenced that sutureless valves when compared to other bioprosthesis have similar 30-day stroke, AKI, major bleeding, PPI, PPM, and post-operative aortic valve area. In the follow-up, we observed a higher risk of mortality (hazard ratio: 1.74; 95% CI: 1.26–2.40; *p* < 0.001) with other bioprosthesis compared to sutureless valves.

The strong points of this study include an update on current publications for sutureless valves and a 360-degree view of the prosthesis when compared to other bioprostheses.

### 5.7. Future Perspectives

SU-AVR have been proven to be a good alternative for old and frail patients undergoing aortic valve replacement. However, SU-AVR has been proven to be a good ‘’marriage’’ in patients undergoing minimally invasive AVR. In this context, this review may contribute to opening a new point of discussion on whether the use of SU-AVR can expand to younger patients, not amenable to aortic valve repair and undergoing minimally invasive SAVR. While patients benefit from minimally invasive cardiac surgery, the procedure itself can be lengthy when compared to traditional AVR. Therefore, the use of a SU-AVR can better suit this patient profile by reducing the duration of the AVR surgical procedure as well as reducing the post-operative complications rate.

## 6. Conclusions

The Perceval bioprosthesis has proved to be a reliable prosthesis for conventional SAVR and mini-SAVR due to its implantation speed, reduced CPB time, reduced AXC time, and shorter intensive care unit and hospital length of stay. In addition, its adoption in hostile roots, and its usage in reinterventions coupled with the low profile render it a formidable tool in the surgical armamentarium. Perceval implantation expectation is zero PVL. Anything above that is likely due to a sub-optimal implant and should be revised. Clearly, this is related to adequate annular debridement and familiarity with optimal implant technique.

## 7. Learning Objectives

What do we already know about the Perceval sutureless valve?

What do we already know about sutureless valves?

1. Sutureless valves have a recognized role in cardiac surgery for aortic valve replacement.

2. Transcatheter aortic valve implantation (TAVR) has emerged as a suitable alternative for aortic valve replacement (AVR).

What does this study add to the literature?

1. SU-AVR surgical indications include (a) patients undergoing cardiac surgery for aortic valve stenosis, (b) mixed valve pathology (stenosis/regurgitation) and (c) reinterventions.

2. SU-AVR have better clinical and echocardiographic outcomes when compared to SB.

3. Instead of adopting the less efficient way of thinking “sutureless better than TAVR or vice versa”, cardiologists should consider the initial pre-interventional risk profile and patient life expectancy when referring patients for these treatments.

## Figures and Tables

**Table 1:** Sutureless aortic valve replacement vs. other stented bioprosthesis.

Study Author	Muneretto et al. [24]		Gilmanov et al. [25]		Pollari et al. [26]		D’Onofrio et al. [27]		Vaquero et al. [28]		Fischlein et al. [29]	
Type of Clinical Study	Prospective		Retrospective		Prospective		Retrospective		Prospective		Prospective	
Valves and patients	Perceval N = 53	Stented N = 55	Perceval N = 133	Stented N = 133	Perceval N = 88	Stented N = 88	Perceval N = 31	Stented N = 112	Perceval N = 140	Stented N = 409	Perceval N = 447	Stented N = 449
30-day Mortality (%)	0	0	0.8	1.5	2.4	3.7	0	1.8	6.4	5.9	1	1
Bleeding requiring surgery (%)	7.5	10.5	6.8	3.8	2.4	6.1	NR	NR	NR	NR	4.4	6.3
Paravalvular leak (%)	1.9	0	NR	NR	NR	NR	19.4	1	3.6	0.5	1	0
Stroke (%)	0	1.8	NR	NR	3.7	7.3	0	0	2.9	2.7	1.5	1.9
Myocardial infarction (%)	0	0	1.5	0	NR	NR	0	0.9	7.8	4.3	1	1.5
Permanent pacemaker implantation (%)	2	1.8	NR	NR	6.1	8.5	3.2	0.9	10.7	2	10.6	3.2
Aortic cross-clamp time in minutes/SD	30.8 ± 13.6	65.3 ± 27.7	56	90	47 ± 16	59 ± 23	NR	NR	65.3 ± 29.1	77.2 ± 30.3	48.5 ± 24.7	65.2 ± 23.6
Cardiopulmonary bypass time in minutes/SD	47 ± 18.5	89.4 ± 20.4	88	120	71 ± 11	92 ± 33	NR	NR	81.3 ± 34.9	95.7 ± 37.9	71.0 ± 34.1	87.8 ± 33.9
Type of stented valves	NA	Perimount, Edwards	NA	CE Edwards, Medtronic, CE standard	NA	NR	NA	NR	NA	Triflecta	NA	NR
**Study author**	**Dalen et al. [30]**				**Forcillo et al. [31]**				**Dokollari et al. [32]**			
**Type of Clinical Study**	**Retrospective**				**Retrospective**				**Retrospective**			
Valves and patients	Perceval = 171		Stented = 171		Perceval = 76		Stented = 319		Perceval = 25		Stented = 57	
30-day Mortality (%)	1.8		2.3		5		6		4		7	
Bleeding requiring surgery (%)	4.1		6.4		8		8		16		15.8	
Paravalvular leak (%)	0		1.2		0		0		NA		NA	
Stroke (%)	2.3		1.2		0		5		7		4	
Myocardial infarction (%)	NR		NR		0		0		10.5		4	
Permanent pacemaker implantation (%)	9.9		2.9		17		8		8.8		4	
Aortic cross-clamp time in minutes/SD	40 ± 15		65 ± 15		46		68		NR		NR	
Cardiopulmonary bypass time in minutes/SD	69 ± 20		87 ± 20		60		85		NR		NR	
Type of stented valves	NA		CE Perimount		NA		CE, Medtronic, Mitroflow, St. Jude epic, St. Jude Biocor		NR		NR	

NA—not applicable, SD—standard deviation, NR—not reported.

**Table 2:** Clinical outcomes of bicuspid aortic valve stenosis treated with sutureless valve.

Study Author	Durdu et al. [33] (Mean ± SD)	Nguyen et al. [34] (Mean ± SD)	Szecel et al. [35] (Mean ± SD)	Miceli et al. [36] (Mean ± SD)	Suri et al. [37] (Mean ± SD)	Dokollari et al. [32]
Number of patients	N = 13 patients	N = 25 patients	N = 11 patients	N = 88 patients	N = 20 patients	N = 25 patients
Type of clinical study	Retrospective	Retrospective	Retrospective	Retrospective	Retrospective	Retrospective
30-day Mortality (%)	0	4	0	1.6	2	0
Bleeding requiring surgery (%)	7.6	1	NR	3.1	4	1
Paravalvular leak (%)	0	0	0	2.3	NR	NR
Stroke (%)	7.6	8	0	4.2	NR	1
Myocardial infarction (%)	0	0	0	NR	NR	0
Permanent pacemaker implantation (%)	7.6	20	0	5.7	NR	2
Aortic cross-clamping time in minutes/SD	40.3 ± 3.1	45.9 ± 14.0	39 ± 13	55	52.3 ± 19.6	NR
Cardiopulmonary bypass time in minutes/SD	54.5 ± 4.4	56.1 ± 14.9	66 ± 22	80	70.2 ± 27.8	NR

NR—not reported, SD—standard deviation.

**Table 3:** Long-term outcomes of the Perceval bioprosthesis.

Late Events > 30 Days	Shrestha et al. [38]	Meuris et al. [39]	Pollari et al. [40]	Dokollari [32]
Studies	N = 729 Patients	N = 30 Patients	N = 547 Patients	N = 101
Type of study	Retrospective	Prospective clinical trial	Retrospective	Retrospective
Follow-up duration	5 years	5 years	8 years	7 years
Deaths (%)	7	28.7	22.5	12.1
Cardiac Deaths (%)	1.4	3.3	NR	5
Valve Explants (%)	1.5	0	NR	NR
Major Paravalvular leak (%)	1	0	NR	1
Endocarditis (%)	1.6	6.6	NR	0
Structural valve deterioration (%)	0	0	4.2	NR
Valve thrombosis (%)	0	0	NR	NR
AV block III (%)	1.4	3.3	NR	3
Stroke	0.8	0		

**Table 4:** Hemodynamic outcomes.

Endpoints	Santarpino et al. [1] N = 658 (Mean ± SD)	Rubino et al. [41] N = 314 (Mean ± SD)	Mazine et al. [42] N = 215 (Mean ± SD)	Folliguet et al. [39] N = 208 (Mean ± SD)	Shrestha et al. [43] N = 30 (Mean ± SD)	Shrestha et al. [44] N = 243 (Mean ± SD)	Miceli et al. [36] N = 37 (Mean ± SD)	Repossini et al. [19] N = 158 (Mean ± SD)
Type of clinical study	Prospective	Retrospective	Retrospective	Retrospective	Prospective	Retrospective	Retrospective	Retrospective
EOA (cm^2^) at discharge	1.5 ± 0.4	NR	1.56 ± 0.37	1.4 ± 0.4	NR	1.5 ± 0.4	NR	NR
EOA (cm^2^) at 6 months	1.5 ± 0.3	NR	NR	1.5 ± 0.4	NR	1.5 ± 0.4	NR	NR
EOA (cm^2^) at 1 year	1.5 ± 0.4	NR	NR	1.5 ± 0.3	1.55 ± 0.35	1.6 ± 0.4	NR	NR
EOA (cm^2^) at 2 years	NR	NR	NR	NR	1.51 ± 0.26	1.7 ± 0.5	NR	NR
Mean gradient (mmHg) at discharge	10.3 ± 4.5	14 ± 6	13.3 ± 6.4	10.4 ± 4.3	NR	10.1 ± 4.7	11.4 ± 3.7	10.9 ± 5.4
Mean gradient (mmHg) at 6 months	8.9 ± 4.1	NR	NR	8.9 ± 3.2	NR	8.9 ± 4.2	NR	NR
Mean gradient (mmHg) at 1 year	9.2 ± 5	NR	NR	8.7 ± 3.7	9.9 ± 4.6	8.9 ± 4.6	NR	NR
Mean gradient (mmHg) at 2 years	NR	NR	NR	NR	8 ± 4.1	9 ± 3.4	NR	NR
Peak gradient (mmHg) at discharge	19.4 ± 8.1	27 ± 11	24.5 ± 10.8	21.3 ± 8.6	NR	20.3 ± 9.9	19.2 ± 6.9	18.7 ± 9.1
Peak gradient (mmHg) at 6 months	16.8 ± 7	NR	NR	19.6 ± 6.7	NR	18 ± 7.6	NR	NR
Peak gradient (mmHg) at 1 year	17.1 ± 8.7	NR	NR	18.8 ± 7.6	20.9 ± 9.2	17.5 ± 8.2	NR	NR
Peak gradient (mmHg) at 2 years	NR	NR	NR	NR	16.6 ± 7.2	18.3 ± 5.6	NR	NR
**Endpoints**		**Chung et al. [23]**	**Suri et al. [37]**	**Durdu et al. [33]**		**Miceli et al. [17]**	**Nguyen et al. [34]**	
Type of clinical study		Retrospective	Retrospective	Retrospective		Retrospective	Retrospective	
EOA (cm^2^) at discharge		1.6 ± 0.4	1.4 ± 0.3	1.81 ± 0.38		NR	1.86 ± 0.6	
EOA (cm^2^) at 6 months		NR	NR	NR		NR	NR	
EOA (cm^2^) at 1 year		1.5 ± 0.3	NR	NR		NR	NR	
EOA (cm^2^) at 2 years		NR	NR	NR		NR	NR	
Mean gradient (mmHg) at discharge		14.7 ± 3.8	10.3 ± 3.7	13.6 ± 4.4		14.8 ± 5.8	12.7 ± 6.4	
Mean gradient (mmHg) at 6 months		NR	NR	NR		NR	NR	
Mean gradient (mmHg) at 1 year		12.4 ± 5.3	NR	NR		NR	NR	
Mean gradient (mmHg) at 2 years		NR	NR	NR		NR	NR	
Peak gradient (mmHg) at discharge		27.5 ± 7.0	NR	NR		28.3 ± 10.9	NR	
Peak gradient (mmHg) at 6 months		NR	NR	NR		NR	NR	
Peak gradient (mmHg) at 1 year		23.8 ± 8.8	NR	NR		NR	NR	
Peak gradient (mmHg) at 2 years		NR	NR	NR		NR	NR	

EOA—effective orifice area; SD—standard deviation; NR—not reported.

**Table 5:** Long-term echocardiographic outcomes (5-year follow-up) of the Perceval bioprosthesis.

Study	Shrestha et al. [45] N = 729 Patients (Mean ± SD)	Meuris et al. [39] N = 30 Patients (Mean ± SD)
LVEF at 3 years (%)	67 ± 9	NR
LVEF at 4 years (%)	66.1 ± 9.1	NR
LVEF at 5 years (%)	65.8 ± 7.7	NR
Mean transvalvular gradient at 3 years mmHg	7.7 ± 2.8	8.3 ± 2.5
Mean transvalvular gradient at 4 years mmHg	7.8 ± 3.8	7.6 ± 3.6
Mean transvalvular gradient at 5 years mmHg	8.8 ± 4.6	9.3 ± 5.5
Peak transvalvular gradient at 3 years mmHg	16 ± 5.2	16.6 ± 6.2
Peak transvalvular gradient at 4 years mmHg	17.8 ± 8.1	17.5 ± 7.8
Peak transvalvular gradients at 5 years mmHg	21.1 ± 9.7	21.4 ± 11.5
EOA at 3 years (cm^2^)	1.64 ± 0.42	1.68 ± 0.4
EOA at 4 years (cm^2^)	1.68 ± 0.43	1.68 ± 0.43
EOA at 5 years (cm^2^)	1.8 ± 0.3	1.69 ± 0.42

**Table 6:** Costs outcomes of the Perceval Valve, TAVR, and Sutured Valves.

Author	Villa et al. [46]	Villa et al. [46]
Study year	2019	2019
Type of study	Retrospective	Retrospective
Type of valve	Perceval	Sutured
Costs in US dollars	12,825	13,543

## Data Availability

Data can be provided upon reasonable request.

## Glossary of Abbreviations

TAVR	transcatheter aortic valve replacement
CPB	cardiopulmonary bypass
PPI	permanent pacemaker implantation
BAV	bicuspid aortic valve
MI	myocardial infarction
PVL	paravalvular leak
SAVR	surgical aortic valve replacement
SU-AVR	sutureless aortic valve replacement
SB	sutured bioprosthesis.

## References

[1] Santarpino G., Pfeiffer S., Concistre G., Grossmann I., Hinzmann M., Fischlein T. (2013). The Perceval S aortic valve has the potential of shortening surgical time: Does it also result in improved outcome?. Ann. Thorac. Surg..

[2] Dokollari A., Ramlawi B., Torregrossa G., Sá M.P., Sicouri S., Prifti E., Gelsomino S., Bonacchi M. (2022). Benefits and Pitfalls of the Perceval Sutureless Bioprosthesis. Front. Cardiovasc. Med..

[3] Dokollari A., Torregrossa G., Sicouri S., Veshti A., Margaryan R., Cameli M., Mandoli G.E., Maccherini M., Montesi G., Cabrucci F. (2022). Pearls, pitfalls, and surgical indications of the Intuity TM heart valve: A rapid deployment bioprosthesis. A systematic review of the literature. J. Card. Surg..

[4] Sá M.P., Jabagi H., Dokollari A., Awad A.K., Van den Eynde J., Malin J.H., Sicouri S., Torregrossa G., Ruhparwar A., Weymann A. (2022). Early and late outcomes of surgical aortic valve replacement with sutureless and rapid-deployment valves versus transcatheter aortic valve implantation: Meta-analysis with reconstructed time-to-event data of matched studies. Catheter. Cardiovasc. Interv..

[5] Leon M.B., Smith C.R., Mack M., Miller D.C., Moses J.W., Svensson L.G., Tuzcu E.M., Webb J.G., Fontana G.P., Makkar R.R. (2010). Transcatheter aortic-valve implantation for aortic stenosis in patients who cannot undergo surgery. N. Engl. J. Med..

[6] Leon M.B., Smith C.R., Mack M.J., Makkar R.R., Svensson L.G., Kodali S.K., Thourani V.H., Tuzcu E.M., Miller D.C., Herrmann H.C. (2016). Transcatheter or Surgical Aortic-Valve Replacement in Intermediate-Risk Patients. N. Engl. J. Med..

[7] Mack M.J., Leon M.B., Thourani V.H., Makkar R., Kodali S.K., Russo M., Kapadia S.R., Malaisrie S.C., Cohen D.J., Pibarot P. (2019). Transcatheter Aortic-Valve Replacement with a Balloon-Expandable Valve in Low-Risk Patients. N. Engl. J. Med..

[8] Reardon M.R., Mieghem N.M.V., Pompa J.J., Kleiman N.S., Sondergaard L., Mumtaz M., Adams D.H., Deeb G.M., Maini B., Gada H. (2017). Surgical or Transcatheter Aortic-Valve Replacement in Intermediate-Risk Patients. N. Engl. J. Med..

[9] Powell R., Pelletier M.P., Chu M.W.A., Bouchard D., Melvin K.N., Adams C. (2017). The Perceval Sutureless Aortic Valve: Review of Outcomes, Complications, and Future Direction. Innovations.

[10] Dokollari A., Cameli M., Mandoli G.E., Kalra D.S., Poston R., Coku L., Pernoci M., Miri M., Bonacchi M., Gelsomino S. (2021). Early and Midterm Clinical Outcomes of Transcatheter Valve-in-Valve Implantation Versus Redo Surgical Aortic Valve Replacement for Aortic Bioprosthetic Valve Degeneration: Two Faces of the Same Medal. J. Cardiothorac. Vasc. Anesth..

[11] Sá M.P., Van den Eynde J., Simonato M., Hirji S., Erten O., Jacquemyn X., Tasoudis P., Dokollari A., Sicouri S., Weymann A. (2023). Late outcomes of valve-in-valve transcatheter aortic valve implantation versus re-replacement: Meta-analysis of reconstructed time-to-event data. Int. J. Cardiol..

[12] Page M.J., McKenzie J.E., Bossuyt P.M., Boutron I., Hoffmann T.C., Mulrow C.D., Shamseer L., Tetzlaff J.M., Akl E.A., Brennan S.E. (2021). The PRISMA 2020 statement: An updated guideline for reporting systematic reviews. BMJ.

[13] Biancari F., Barbanti M., Santarpino G., Deste W., Tamburrino C., Gulino S., Immè S., Di Simone E., Todaro D., Pollari F. (2016). Immediate outcome after sutureless versus transcatheter aortic valve replacement. Heart Vessels.

[14] Muneretto C., Solinas M., Folliguet T., Di Bartolomeo R., Repossini A., Laborde F., Rambaldini M., Santarpino G., Di Bacco L., Fischlein T. (2020). Sutureless versus transcatheter aortic valves in elderly patients with aortic stenosis at intermediate risk: A multi-institutional study. J. Thorac. Cardiovasc. Surg..

[15] D’Onofrio A., Rizzoli G., Messina A., Alfieri O., Lorusso R., Salizzoni S., Glauber M., Di Bartolomeo R., Besola L., Rinaldi M. (2013). Conventional surgery, sutureless valves, and transapical aortic valve replacement: What is the best option for patients with aortic valve stenosis? A multicenter, propensity-matched analysis. J. Thorac. Cardiovasc. Surg..

[16] Santarpino G., Pfeiffer S., Jessl J., Dell’Aquila A.M., Pollari F., Pauschinger M., Fischlein T. (2014). Sutureless replacement versus transcatheter valve implantation in aortic valve stenosis: A propensity matched analysis of 2 strategies in high-risk patients. J. Thorac. Cardiovasc. Surg..

[17] Miceli A., Gilmanov D., Murzi M., Marchi F., Ferrarini M., Cerillo A.G., Quaini E., Solinas M., Berti S., Glauber M. (2016). Minimally invasive aortic valve replacement with a sutureless valve through a right anterior mini-thoracotomy versus transcatheter aortic valve implantation in high-risk patients. Eur. J. Cardiothorac. Surg..

[18] Muneretto C., Bisleri G., Moggi A., Di Bacco L., Tespili M., Repossini A., Rambaldini M. (2014). Interact Cardiovasc Thorac Surg. Treating the patients in the ‘grey-zone’ with aortic valve disease: A comparison among conventional surgery, sutureless valves and transcatheter aortic valve replacement. Interact. Cardiovasc. Thorac. Surg..

[19] Repossini A., Fischlein T., Solinas M., Di Bacco L., Passaretti B., Grubitzsch H., Folliguet T., Santarpino G., Laborde F., Muneretto C. (2018). Stentless sutureless and transcatheter valves: A comparison of the hemodynamic performance of different prostheses concept. Minerva Cardioangiol..

[20] Gerfer S., Mauri V., Kuhn E., Adam M., Djordevic I., Ivanov B., Gaisendrees C., Frerker C., Schmidt T., Mader N. (2021). Comparison of Self-Expanding RDV Perceval S versus TAVI ACURATE neo/TF. Thorac. Cardiovasc. Surg..

[21] Zubarevich A., Szczechowicz M., Amanov L., Arjomandi Rad A., Osswald A., Torabi S., Ruhparwar A., Weymann A. (2022). Non-Inferiority of Sutureless Aortic Valve Replacement in the TAVR Era: David versus Goliath. Life.

[22] Vilalta V., Alperi A., Cediel G., Mohammadi S., Fernández-Nofrerias E., Kalvrouziotis D., Delarochellière R., Paradis J.M., González-Lopera M., Fadeuilhe E. (2021). Midterm Outcomes Following Sutureless and Transcatheter Aortic Valve Replacement in Low-Risk Patients With Aortic Stenosis. Circ. Cardiovasc. Interv..

[23] Chung Y.H., Lee S.H., Ko Y.G., Lee S., Shim C.Y., Ahn C.M., Hong G.R., Shim J.K., Kwak Y.L., Hong M.K. (2021). Transcatheter Aortic Valve Replacement versus Sutureless Aortic Valve Replacement: A Single Center Retrospective Cohort Study. Yonsei Med. J..

[24] Santarpino G., Lorusso R., Moscarelli M., Mikus E., Wisniewski K., Dell’Aquila A.M., Margari V., Carrozzo A., Barbato L., Fiorani V. (2022). Sutureless versus transcatheter aortic valve replacement: A multicenter analysis of “real-world” data. J. Cardiol..

[25] Muneretto C., Alfieri O., Cesana B.M., De Bonis M., Di Bartolomeo R., Savini C., Folesani G., Di Bacco L., Rambaldini M., Maureira J.P. (2015). A comparison of conventional surgery, transcatheter aortic valve replacement, and sutureless valves in “real-world” patients with aortic stenosis and intermediate- to high-risk profile. J. Thorac. Cardiovasc. Surg..

[26] Gilmanov D., Miceli A., Ferrarini M., Farneti P., Murzi M., Solinas M., Glauber M. (2014). Aortic valve replacement through right anterior minithoracotomy: Can sutureless technology improve clinical outcomes?. Ann. Thorac. Surg..

[27] Pollari F., Santarpino G., Dell’Aquila A.M., Gazdag L., Alnahas H., Vogt F., Pfeiffer S., Fischlein T. (2014). Better short-term outcome by using sutureless valves: A propensity-matched score analysis. Ann. Thorac. Surg..

[28] D’Onofrio A., Messina A., Lorusso R., Alfieri O., Fusari M., Rubino P., Rinaldi M., Di Bartolomeo R., Glauber M., Troise G. (2012). Sutureless aortic valve replacement as an alternative treatment for patients belonging to the “gray zone” between transcatheter aortic valve implantation and conventional surgery: A propensity-matched, multicenter analysis. J. Thorac. Cardiovasc. Surg..

[29] Fischlein T., Folliguet T., Meuris B., Shrestha M.L., Roselli E.E., McGlothlin A., Kappert U., Pfeiffer S., Corbi P., Lorusso R. (2021). Sutureless versus conventional bioprostheses for aortic valve replacement in severe symptomatic aortic valve stenosis. J. Thorac. Cardiovasc. Surg..

[30] Dalen M., Biancari F., Rubino A.S., Santarpino G., Glaser N., Praetere H.D., Kasama K., Juvonen T., Deste W., Pollari F. (2016). Aortic valve replacement through full sternotomy with a stented bioprosthesis versus minimally invasive sternotomy with a sutureless bioprosthesis. Eur. J. Cardithorac. Surg..

[31] Forcillo J., Bouchard D., Nguyen A., Perrault L., Cartier R., Pellerin M., Demers P., Stevens L.M., Carrier M. (2016). Perioperative outcomes with sutureless versus stented biological aortic valves in elderly persons. J. Thorac. Cardiovasc. Surg..

[32] Dokollari A., Margaryan R., Torregrossa G., Sicouri S., Cameli M., Mandoli G.E., Prifti E., Veshti A., Bonacchi M., Gelsomino S. (2023). Risk predictors that impact long-term prognosis in patients undergoing aortic valve replacement with the Perceval sutureless bioprosthesis. Cardiovasc. Revasc. Med..

[33] Durdu M.S., Gumus F., Ozcinar E., Cakici M., Bermede O., Dincer I., Kılıckap M., Sirlak M., Ucanok K., Akar A.R. (2019). Sutureless Valve Replacement Through a Right Anterior Mini-thoracotomy in Elderly Patients With Stenotic Bicuspid Aortic Valve. Semin. Thorac. Cardiovasc. Surg..

[34] Nguyen A., Fortin W., Mazine A., Bouchard D., Carrier M., El-Hamamsy I., Lamarche Y., Demers P. (2015). Sutureless aortic valve replacement in patients who have bicuspid aortic valve. J. Thorac. Cardiovasc. Surg..

[35] Szecel D., Eurlings R., Rega F., Verbrugghe P., Meuris B. (2020). Perceval sutureless aortic valve implantation: Mid-term outcomes. Ann. Thorac. Surg..

[36] Miceli A., Berretta P., Fiore A., Andreas M., Solinas M., Santarpino G., Kappert U., Misfeld M., Savini C., Albertini A. (2020). Sutureless and rapid deployment implantation in bicuspid aortic valve: Results from the sutureless and rapid-deployment aortic valve replacement international registry. Ann. Cardiothorac. Surg..

[37] Suri R.M., Javadikasgari H., Heimansohn D.A., Weissman N.J., Ailawadi G., Ad N., Aldea G.S., Thourani V.H., Szeto W.Y., Michler R.E. (2019). Prospective USinvestigational device exemption trial of a sutureless aorticbioprosthesis: One-year outcomes. J. Thorac. Cardiovasc. Surg..

[38] Shrestha M., Fischlein T., Meuris B., Flameng W., Carrel T., Madonna F., Misfeld M., Folliguet T., Haverich A., Laborde F. (2016). European multicentre experience with the sutureless Perceval valve: Clinical and haemodynamic outcomes up to 5 years in over 700 patients. Eur. J. Cardiothorac. Surg..

[39] Meuris B., Flameng W.J., Laborde F., Folliguet T.A., Haverich A., Shrestha M. (2015). Five-year results of the pilot trial of a sutureless valve. J. Thorac. Cardiovasc. Surg..

[40] Pollari F., Mamdooh H., Hitzl W., Grossmann I., Vogt F., Fischlein T. (2023). Ten years’ experience with the sutureless aortic valve replacement: Incidence and predictors for survival and valve durability at follow-up. Eur. J. Cardiothorac. Surg..

[41] Rubino A.S., Mignosa C. (2018). Sutureless valves and the quality of perfusion: Towards a goal directed aortic valve replacement. Minerva. Cardioangiol..

[42] Mazine A., Teoh K., Bouhout I., Bhatnagar G., Pelletier M., Voisine P., Demers P., Carrier M., Bouchard D. (2015). Sutureless aortic valve replacement: A Canadian multicentre study. Can. J. Cardiol..

[43] Shrestha M., Folliguet T., Meuris B., Dibie A., Bara C., Herregods M.C., Khaladj N., Hagl C., Flameng W., Laborde F. (2009). Sutureless Perceval S aortic valve replacement: A multicenter, prospective pilot trial. J. Heart Valve Dis..

[44] Shrestha M., Maeding I., Höffler K., Koigeldiyev N., Marsch G., Siemeni T., Fleissner F., Haverich A. (2013). Aortic valve replacement in geriatric patients with small aortic roots: Are sutureless valves the future?. Interact. Cardiovasc. Thorac. Surg..

[45] Villa E., Dalla Tomba M., Messina A., Trenta A., Brunelli F., Cirillo M., Mhagna Z., Chiariello G.A., Troise G. (2019). Sutureless aortic valve replacement in high risk patients neutralizes expected worse hospital outcome: A clinical and economic analysis. Cardiol. J..

[46] Sá M.P., Sun T., Fatehi Hassanabad A., Awad A.K., Van den Eynde J., Malin J.H., Sicouri S., Torregrossa G., Ruhparwar A., Weymann A. (2022). Complete transcatheter versus complete surgical treatment in patients with aortic valve stenosis and concomitant coronary artery disease: Study-level meta-analysis with reconstructed time-to-event data. J. Card. Surg..

[47] Povero M., Miceli A., Pradelli L., Ferrarini M., Pinciroli M., Glauber M. (2018). Cost-utility of surgical sutureless bioprostheses vs TAVI in aortic valve replacement for patients at intermediate and high surgical risk. Clin. Outcomes Res..

[48] Sá M.P., Jacquemyn X., Van den Eynde J., Tasoudis P., Dokollari A., Torregrossa G., Sicouri S., Clavel M.A., Pibarot P., Ramlawi B. (2023). Impact of Prosthesis-Patient Mismatch After Transcatheter Aortic Valve Replacement: Meta-Analysis of Kaplan-Meier-Derived Individual Patient Data. JACC Cardiovasc. Imaging.

[49] Deeb G.M., Reardon M.J., Ramlawi B., Yakubov S.J., Chetcuti S.J., Kleiman N.S., Mangi A.A., Zahr F., Song H.K., Gada H. (2022). Propensity-Matched 1-Year Outcomes Following Transcatheter Aortic Valve Replacement in Low-Risk Bicuspid and Tricuspid Patients. JACC Cardiovasc. Interv..

[50] Bedeir K., Reardon M., Cohn L.H., Ramlawi B. (2016). Sutureless Aortic Valves: Combining the Best or the Worst?. Semin. Thorac. Cardiovasc. Surg..

[51] Dokollari A., Sá M.P., Sicouri S., Ramlawi B., Torregrossa G., Bonacchi M. (2022). Commentary: Osteogenic Metaplasia of the Aortic Valve. Do Bacteria, Diabetes, and Dyslipidemia Play a Role?. Semin. Thorac. Cardiovasc. Surg..

[52] Bonacchi M., Dokollari A., Parise O., Sani G., Prifti E., Bisleri G., Gelsomino S. (2021). Ministernotomy compared with right anterior minithoracotomy for aortic valve surgery. J. Thorac. Cardiovasc. Surg..

[53] Prifti E., Bonacchi M., Minardi G., Krakulli K., Baboci A., Esposito G., Demiraj A., Zeka M., Rruci E. (2018). Early and Mid-term Outcome of the St. Jude Medical Regent 19-mm Aortic Valve Mechanical Prosthesis. Functional and Haemodynamic Evaluation. Heart Lung Circ..

[54] Bach D.S., Kon N.D. (2014). Long-term clinical outcomes 15 years after aortic valve replacement with the Freestyle stentless aortic bioprosthesis. Ann. Thorac. Surg..

[55] Kim M.S., Kim J.H., Joo H.C., Lee S., Youn Y.N., Lee S.H. (2020). Prognostic Markers and Long-Term Outcomes After Aortic Valve Replacement in Patients with Chronic Aortic Regurgitation. J. Am. Heart Assoc..

